# Proteins in Tumor-Derived Plasma Extracellular Vesicles Indicate Tumor Origin

**DOI:** 10.1016/j.mcpro.2022.100476

**Published:** 2022-12-05

**Authors:** Meltem Barlin, Petra Erdmann-Gilmore, Jacqueline L. Mudd, Qiang Zhang, Robert W. Seymour, Zhanfang Guo, Julia R. Miessner, S. Peter Goedegebuure, Ye Bi, Omar A. Osorio, Jennifer Alexander-Brett, Shunqiang Li, Cynthia X. Ma, Ryan C. Fields, R. Reid Townsend, Jason M. Held

**Affiliations:** 1 Department of Medicine, Washington University School of Medicine in St Louis, St Louis, Missouri, USA; 2 Siteman Cancer Center, Washington University School of Medicine in St Louis, St Louis, Missouri, USA; 3 Department of Surgery, Washington University School of Medicine in St Louis, St Louis, Missouri, USA; 4 Department of Pathology and Immunology, Washington University School of Medicine in St Louis, St Louis, Missouri, USA; 5 Department of Anesthesiology, Washington University School of Medicine in St Louis, St Louis, Missouri, USA

**Keywords:** exosomes, extracellular vesicles, proteomics, patient-derived xenografts, cancer, ProteoClade, machine learning, CRC, colorectal cancer, DB, digestion buffer, DLS, dynamic light scattering, EV, extracellular vesicle, FA, formic acid, LDA, linear discriminant analysis, LFQ, label-free quantitation, MS, mass spectrometry, PDAC, pancreatic ductal carcinoma, PDX, patient-derived xenograft, RT, room temperature

## Abstract

Cancer-derived extracellular vesicles (EVs) promote tumorigenesis, premetastatic niche formation, and metastasis *via* their protein cargo. However, the proteins packaged by patient tumors into EVs cannot be determined *in vivo* because of the presence of EVs derived from other tissues. We therefore developed a cross-species proteomic method to quantify the human tumor-derived proteome of plasma EVs produced by patient-derived xenografts of four cancer types. Proteomic profiling revealed individualized packaging of novel protein cargo, and machine learning accurately classified the type of the underlying tumor.

## Highlights

• A cross-species profile of the proteins PDXs package into extracellular vesicles.
• About 0.02 to 0.05% of proteins in plasma extracellular vesicles are derived from the tumor.
• Protein packaging into EVs is an intrinsic property of each tumor.
• Machine learning predicted the underlying tumor type with more than 92% accuracy.

## Graphical Abstract

**Figure:**
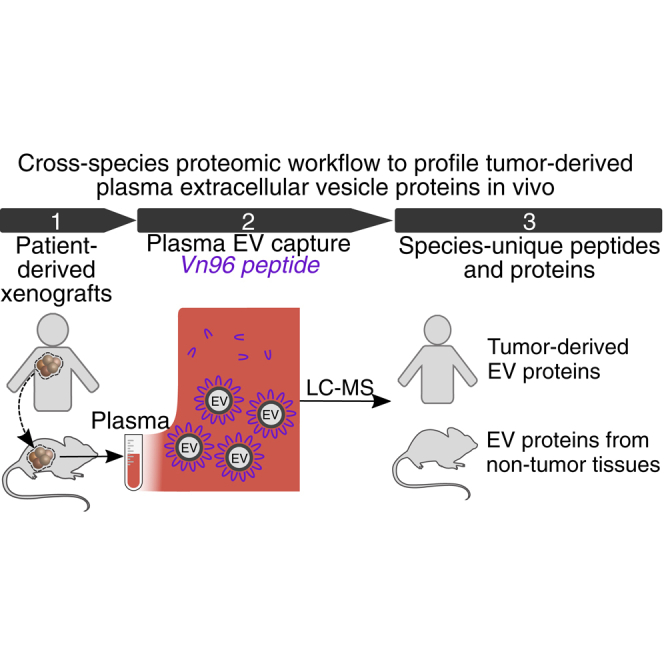

## In Brief

We report of a new cross-species proteomic approach to profile which proteins patient-derived xenograft tumors package into plasma extracellular vesicles *in vivo*. We find that 0.02 to 0.05% of proteins in plasma extracellular vesicles are derived from the tumor, with some proteins common to all four cancer models evaluated and some proteins expressed uniquely in certain cancer types. Machine learning classification can predict the underlying tumor type with greater than 92% accuracy.

Plasma extracellular vesicles (EVs) play a functional role in cell–cell signaling, and their bioactive cargoes are sentinels of organismal homeostasis. Since EVs can be readily assayed by noninvasive liquid biopsies, they also have significant potential for disease diagnosis, prognosis, and surveillance, as well as investigating the molecular mechanisms of extracellular communication. In cancer, tumor-derived EVs facilitate premetastatic niche formation and metastasis *via* their protein cargo, which can remodel the extracellular matrix, regulate stromal cell differentiation, alter vascular permeability, suppress immune cells, and guide organotropic metastasis (1, 2, 3).

New insights into the EV biology of tumors *in vivo* are limited by the release of EVs by the other tissues in the body. These tumor-independent EVs create a high background, which confounds unambiguous determination of which EV proteins are tumor derived. For this reason, studies typically utilize EVs generated *in vitro* by cell lines for *in vivo* analysis (2). Alternatively, studies analyzing the plasma EV cargo of cancer patients may identify putative cancer biomarkers, but this approach cannot verify that the altered cargo is tumor derived. This caveat is especially relevant when the goal is identifying biomarkers of tumor treatment response since anticancer therapy also effects nontumor tissues and likely impacts stromal EV biology.

Proteomic profiling of EVs has greatly improved our understanding of EV cargo by providing a more complete and nuanced understanding of EV biology and markers (4). Proteomic analysis facilitated a recent reassessment of exosome composition and discovered that annexin A1 is a specific marker for EVs shed from the plasma membrane (5). Proteomic profiling also contributed to the recent discovery of exomeres, an exosomal subpopulation of nonmembranous nanoparticles approximately 35 nm in size (6). Since there are no agreed upon protein markers present or absent in all EV subpopulations (7), global protein profiling provides a more complete picture of EVs than candidate approaches such as Western blotting.

The chemical information captured by mass spectrometry (MS)–based proteomic technologies can be coupled with bioinformatics to uniquely answer a range of important biological questions. For example, stable isotope–labeled amino acids can be used to encode quantitative information about protein expression (8, 9), chemical labeling (10), or investigating aspects of protein biochemistry such as protein turnover (11). Mass shifts can indicate the presence and position of post-translational modifications (12). In addition, the amino acid sequence information provided by MS can be used to deconvolute the proteomes of multiple species present in a single location or sample. This cross-species proteomic approach has broad applications for metaproteomics (13), but it has also been used to investigate cancer xenograft models where human-specific peptide and protein sequences are derived from the tumor and murine-specific sequences are from the mouse stroma. This approach has been used to investigate how tumors educate the stroma (14) and the integrated response of the tumor and stroma to anticancer drugs (15). One of the biggest challenges of cross-species analysis is bioinformatically assigning taxon-specific peptides, but several software tools have been recently developed to facilitate species-specific deconvolution (16, 17).

In the present study, we utilized cancer patient–derived xenograft (PDX) models to determine which proteins are packaged by patient tumors *in vivo* into EVs using an approach that integrates plasma EV enrichment, cross-species proteomics, and machine learning–based classification. We proteomically profiled 14 PDX across four cancer types (supplemental Table S1), and tumors were derived from a mix of primary tumors and those of metastatic origin in the patients. The method uses a commercial EV isolation kit and open source software tools to streamline and maximize its accessibility to investigate many aspects of tumor EV biology *in vivo*.

## Experimental Procedures

### Experimental Design and Statistical Rationale

#### PDX Generation and Growth

Animal studies were performed under an approved animal studies protocol at the Washington University School of Medicine. Sample N and biological replicates are described in supplemental Table S1.

For breast PDX generation, female homozygous nude mice (Charles River Laboratories; catalog no.: 088) were injected with 1 × 10^6^ patient-derived breast tumor cells mixed with an equal volume of Matrigel media (BD Biosciences; catalog no.: 354234) and 10% fetal bovine serum (Fisher Scientific; catalog no.: Mt35010CV) in RPMI (Fisher Scientific; catalog no.: SH30027LS) into the fourth mammary fat pad.

Pancreatic ductal carcinoma (PDAC), colorectal cancer (CRC), and melanoma PDXs were generated in female NSG NOD.Cg-Prkdc^scid^ IL2rg^tm1Wjil^/SzJ mice (Jackson Laboratory; catalog no.: 005557). To establish tumor growth, mice were anesthetized with isoflurane, a subcutaneous nick was made in each flank, and a small tumor fragment coated with Matrigel was transferred into each subcutaneous pocket (implanted tumor size: 1 mm × 1 mm × 1 mm). Nicks were closed with a small amount of GLUture topical adhesive.

#### Plasma Collection

Avertin (Sigma–Aldrich; catalog no.: T48402-25G) at a dose of 500 to 1000 mg/kg or ketamine (100 mg/kg) plus xylazine (10 mg/kg) was given intraperitoneally for cardia puncture blood collection with either 25G needles (Fisher Scientific; catalog no.: 14-829-2C) or 26G needles (Fisher Scientific; catalog no.: 14-823-2E). At least 200 μl of peripheral blood was collected in K3 EDTA tubes (Sarstedt; catalog no.: 41.1504.105), centrifuged at 1.5 to 2*g*, 4 °C for 10 min, and immediately transferred into Nalgene Cryogenic Tubes at room temperature (RT; Thermo Fisher Scientific; catalog no.: 5000-1020) and stored at −80 °C. Plasma was collected from PDX models no sooner than the second serial passage to minimize the influence of residual nondividing and nontumorigenic stroma on analysis.

#### EV Preparation

EV capture was performed using the ME kit from New England Peptide. Manufacturer instructions were followed with the following modifications. Aliquots of frozen mouse plasma were thawed on ice, vortexed, and spun at 15,000*g* for 10 min. About 200 μl of plasma was added to a new 1.7 ml microcentrifuge tube (Axygen; catalog no.: MCT-175-C) containing 200 μl of PBS (Gibco; catalog no.: 370011044) (10×, pH 7.4; diluted to 1× with LC–MS grade water) and mixed by gentle inversion. EV precipitation was initiated by addition of 8 μl Vn96 peptide stock (prepared per manufacturer instructions) followed by mixing *via* inversion and incubation at RT with end-over-end rotation for 1 h. Samples were centrifuged at 17,000*g* for 15 min at 4 °C to collect the EVs at the bottom of the tube. The supernatant was carefully removed, and the pellet was resuspended in 400 μl of PBS. The samples were spun again at 17,000*g* for 15 min at 4 °C. The EV pellet was washed two more times with PBS as described previously. After the last spin, the pellets were stored at −80 °C until peptide preparation. All steps were performed at RT unless otherwise noted. The default brake settings of the centrifuge were used.

#### EV Size Analysis

Dynamic light scattering (DLS) was used to analyze size distribution of EVs isolated from mouse serum. Following precipitation with Vn96 peptide, the pellet was solubilized in PBS/8% trehalose (from 30% w/v trehalose dihydrate stock; Hampton Research) with incubation in a Branson B200 ultrasonic bath for 10 min. Sample was then centrifuged at 14,000*g* and analyzed on a Malvern NanoS instrument at 25 °C. A representative intensity profile is shown in supplemental Fig. S1. Cumulant size (intensity-weighted Z-average) was reproducible between runs, and polydispersity was low, which indicated a relatively homogeneous distribution of particles. These results are consistent with measurements from cell line–derived EVs purified by size-exclusion chromatography and validated by transmission electron microscopy (18).

#### Peptide Preparation

Samples were digested as previously described (19, 20) using a modification of the filter-aided sample preparation method (21). The EV pellets were solubilized with 30 μl SDS buffer (4% [w/v] SDS, 100 mM Tris–HCl [pH 8.0]). The samples were reduced by addition of 50 mM DTT with heating to 95 °C for 10 min. The reduced samples were mixed with 200 μl of 100 mM Tris–HCl buffer, pH 8.5 containing 8 M urea (UA buffer), transferred on top chamber of a 30,000 molecular weight cutoff filtration unit (Millipore; catalog no.: MRCF0R030), and spun in a microcentrifuge at 14,000*g* for 10 min. An additional 200 μl of 100 mM Tris–HCl buffer, pH 8.5, containing 8 M urea (UA buffer) was added to the top chamber of the filter unit, and the filter was spun at 14,000*g* for 15 to 20 min in a microcentrifuge (Eppendorf 5424; Eppendorf, catalog no.: 2231000767). The flow through was discarded, and the proteins were alkylated by addition of 100 μl of 50 mM iodoacetamide (Pierce; catalog no.: A39271) in UA buffer to the top chamber of the filtration unit and gyrating at 550 rpm in the dark at RT for 30 min using a thermomixer (Thermomixer R; Eppendorf). The filter was spun at 14,000*g* for 15 min, and the flow through was discarded. Unreacted iodoacetamide was washed through the filter with two sequential additions of 200 μl of 100 mM Tris–HCl buffer, pH 8.5 containing 8 M urea, and centrifuged at 14,000*g* for 15 to 20 min after each addition. The urea buffer was exchanged into digestion buffer (DB), 50 mM ammonium bicarbonate buffer, pH 8. Two sequential additions of DB (200 μl) with centrifugation after each addition to the top chamber were performed. The top filter units were transferred to a new collection tube, 100 μl DB containing 1 μAU of LysC (Wako Chemicals; catalog no.: 129-02541) was added, and samples were digested at 37 °C. After 2 h of LysC digestion, 1 μg of sequencing-grade trypsin (Promega; catalog no.: V5113) was added, and samples were digested overnight at 37 °C. The filters were spun at 14,000*g* for 15 min to collect the peptides in the flow through. The filter was washed with 50 μl 100 mM ammonium bicarbonate buffer, and the wash was collected with the peptides. In preparation for desalting, peptides were acidified to pH 2 with 1% (v/v) TFA. The peptides were desalted using two microtips (porous graphite carbon; Glygen BIOMEKNT3CAR) on a Beckman robot (Biomek NX), as previously described (22). The peptides were eluted with 60 μl of 60% (v/v) acetonitrile in 0.1% TFA (v/v) and dried in a Speed-Vac (Thermo Fisher Scientific, model no.: Savant DNA 120 concentrator) after adding TFA to 5% (v/v). The peptides were dissolved in 20 μl of 1% (v/v) acetonitrile in water. An aliquot (10%) was removed for quantification using the Pierce Quantitative Fluorometric Peptide Assay kit (Thermo Fisher Scientific; catalog no.: 23290). The remaining peptides were transferred to autosampler vials (Sun-Sri; catalog no.: 200046), dried, and stored at −80 °C for LC–MS analysis. The average peptide yield across samples was 31.5 μg per ml of plasma.

#### Lipid Quantitation

Lipid quantitation was performed using the SPV assay per the manufacturer’s instructions (Cell Biolabs; catalog no: STA-613). The average lipid yield across samples was 59.6 μg per ml of plasma, and the average peptide:lipid ratio was 0.53.

#### *Nano*LC–MS/MS

The samples in formic acid (FA; 1%) were loaded (2.5 μl) onto a 75 μXm i.d. × 50 cm Acclaim PepMap 100 C18 RSLC column (Thermo Fisher Scientific) on an EASY *nano*LC (Thermo Fisher Scientific) at a constant pressure of 700 bar at 100% mobile phase A (1% FA). Prior to sample loading, the column was equilibrated to 100% mobile phase A for a total of 11 μl at 700 bar pressure. Peptide chromatography was initiated with mobile phase A (1% FA) containing 2% mobile phase B (100% acetonitrile [MeCN] and 1% FA) for 5 min, then increased to 20% B over 100 min, to 32% B over 20 min, to 95% B over 1 min and held at 95% B for 19 min, with a flow rate of 250 nl/min. The data were acquired in data-dependent acquisition mode. The full scan mass spectra were acquired with a Q-Exactive mass analyzer with a scan range of *m/z* = 325 to 1500 and a mass resolving power set to 70,000. Ten data-dependent high-energy collisional dissociations were performed with a mass resolving power set to 17,500, a fixed lower value of *m/z* 100, an isolation width of 2 Da, and a normalized collision energy setting of 27. The maximum injection time was 60 ms for parent-ion analysis and product-ion analysis. The target ions that were selected for MS/MS were dynamically excluded for 15 s. The automatic gain control was set at a target value of 1e6 ions for full MS scans and 1e5 ions for MS2. Peptide ions with charge states of 1 or >8 were excluded for high-energy collisional dissociation acquisition.

#### Protein Identification

LC–MS data were searched against MaxQuant (https://www.maxquant.org/) search engine (23) (version 1.6.17.0). MaxQuant was set to search against a concatenated UniProt (version March 2020) database of human (20,365 entries), mouse (17,033 entries), and common contaminant proteins (cRAP; version 1.0; January 1, 2012; 116 entries). Enzyme cleavage specificity was trypsin/P with a maximum of four missed cleavages allowed. The MS2 database searches were performed with a fragment ion mass tolerance of 20 ppm and a parent ion tolerance of 20 ppm. Carbamidomethylation of cysteine was specified in MaxQuant as a fixed modification. Deamidation of asparagine, formation of pyroglutamic acid from N-terminal glutamine, acetylation of protein N terminus, oxidation of methionine, and pyrocarbamidomethylation of N-terminal cysteine were specified as variable modifications. Peptides and proteins were filtered at 1% false discovery rate by searching against a reversed database. Peptides not detected in all three replicates of at least two samples were filtered out to ensure robust detection. The ratios of peptides were calculated in relative to the MaxQuant label-free quantitation (LFQ) intensities of peptides across all samples (24), and the medians were taken to represent protein ratios. The ratios of peptides and proteins were then transformed such that the median under each sample was zero on a log_2_ scale. The offsets used in the median centering of peptide and protein ratios were applied to scale intensity values accordingly.

#### LC–MS Data Analysis

**Fig. 1:**
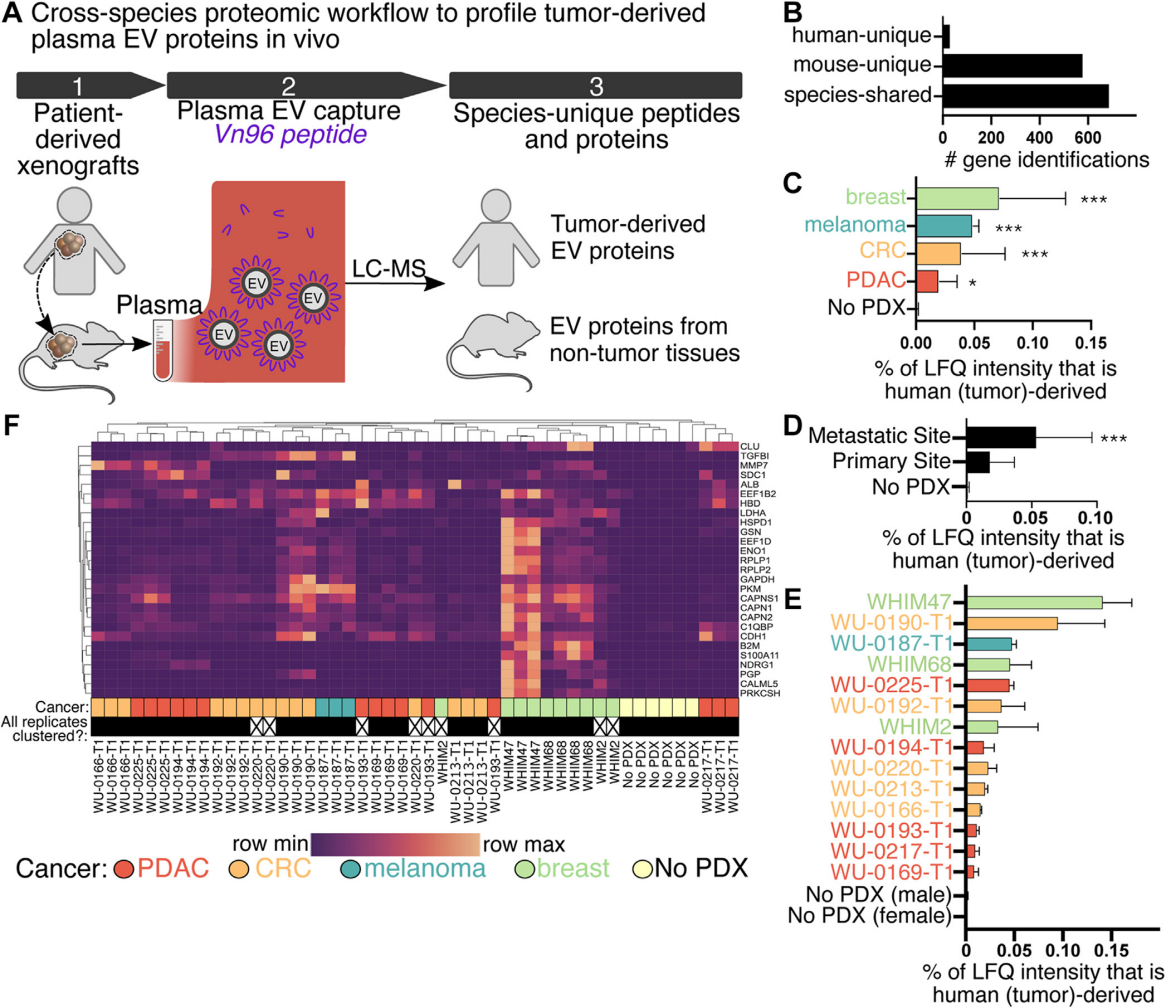
**Patient tumor–derived EV proteins are an intrinsic property of the individual patient tumor and indicative of the tumor type and metastatic origin.***A*, overview schematic of cross-species proteomic profiling of tumor-derived plasma EV proteins. *B*, number of species-specific assignments. *C*, total human-unique (tumor-derived) protein intensity by tumor type, (*D*) tumor site, and (*E*) individual PDX lines. *F*, heatmap of tumor-derived proteins across PDX lines and biological replicates. *Filled black squares* indicate each biological replicates clustered together. ∗*p* < 0.05, ∗∗∗*p* <0.005, based on Kruskal–Wallis one-way ANOVA, and N is detailed in supplemental Table S1. EV, extracellular vesicle; PDX, patient-derived xenograft.

**Fig. 2:**
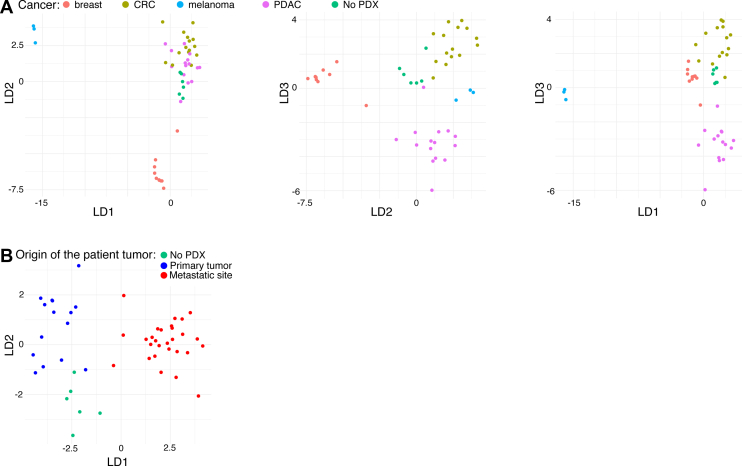
**Tumor classification *via* the tumor-derived EV proteome using machine learning.***A*, LDA classification of PDXs based on tumor type or (*B*) origin of the patient tumor. EV, extracellular vesicle; LDA, linear discriminant analysis; PDX, patient-derived xenograft.

ProteoClade (16) was used to assign peptides as human unique, mouse unique, or species shared. Species-shared peptides were removed for analysis since the tissue origin is ambiguous. Genes assigned by detection of only a single gene–unique peptide sequence were confirmed and required to have either been detected by (1) either top four most observed peptides in the gene in the PeptideAtlas database (25) or (2) at least 5000 total observations of the peptide in PeptideAtlas. The MS1 signal of human peptides was quantified using Skyline (26) for the most accurate LFQ results. Peptides with low signal intensity or interferences were removed, as previously described (10). Skyline MS1 quantitation was used in Figures 1, *E*, *F*, and 2. The heatmap was generated with the Seaborn package using default parameters with Python 3.9.6. Genes were matched to the ExoCarta database (27) for assignment in supplemental Table S2.

Linear discriminant analysis (LDA) and machine learning were performed using both Python 3.9 and R 4.1. Human protein expression was first standardized by mean centering and scaled to unit variance using the StandardScalar function of scikit-learn 1.0. The LDA plot was generated using the MASS package (28), and classification was performed using Auto-SKlearn 2.0 (29) with only the LDA classifier as detailed in the code supplement. About 25% of the dataset was held out for validation and not used in model generation, and accuracy was determined using the sklearn.metrics.accuracy_scoring function.

## Results

### VN96 Captures EV Subpopulation Ranging from Exomeres to Microvesicles

Our approach to identify *bona fide* human proteins packaged into plasma EVs by patient tumors *in vivo* utilizes cross-species proteomic profiling of plasma EVs captured with the VN96 peptide (30) in patient-derived xenograft (PDX) mouse models (Fig. 1*A*). After parsing the amino acid sequence of the identified peptides by species (16), human-unique peptide sequences detected in EVs verify the protein’s origin to be the tumor and not the stroma or secondary mouse tissues (14). At least three biological replicates of 14 PDX lines were profiled. PDXs were generated from a mix of primary tumors and those of metastatic origin in the patient across four cancer types: breast, PDAC, CRC, and melanoma (supplemental Table S1). Tumor attributes are listed in supplemental Table S1. Six nontumor-bearing “no PDX” mice were assayed as controls.

The size of isolated plasma EVs enriched by the VN96 EV capture technology was determined by DLS. A major advantage to DLS is that it samples a broad range of vesicle sizes (1–10,000 nm diameter), which may be present in complex specimens. Most EVs were between 35 and 400 nm with a peak near 165 nm (supplemental Fig. S1). This size distribution indicated robust enrichment of all EV subpopulations including exomeres, exosomes, and microvesicles but not larger apoptotic bodies or intact cells (5). It is possible that some EVs are not precipitated *via* the VN96 method, but prior studies have found that the proteome of VN96-enriched EVs is similar to those isolated by ultracentrifugation (31).

Proteomic profiling of the enriched EVs assigned 5556 peptides with at least six peptide spectral matches below a 1% false discovery rate across the dataset to 1001 genes (supplemental Tables S1, S2, and supplemental Fig. S2). Common contaminant proteins were filtered out using the CRAPome (Contaminant Repository for Affinity Purification) dataset, such as keratins, which could be introduced by sample handling (32). However, human albumin was not filtered out, as discussed later.

About 65.1% of the identified peptide sequences were mouse unique, and 1.0% were human unique based on cross-species parsing with ProteoClade (16). With regard to gene assignment, 44.6% of peptides were uniquely assigned to mouse, whereas 2.2% were uniquely assigned to human genes (Fig. 1*B*). Consistent with size analysis, many common EV markers were robustly detected using mouse or species-shared peptides including HSPA5 (BIP), PDCD6IP (ALIX), and HSPA8 (supplemental Table S2). In addition, we detected markers of specific subpopulations such as ANXA1, a specific indicator of EVs shed from the plasma membrane (5), as well as exomere markers including MTHFD1 and IHD1 (6).

### The Tumor-Derived EV Proteome Varies by Cancer Type and Individual PDX

Quantitation of species-specific peptides was performed using the MS1 precursor intensities determined by LFQ. Across all 14 PDX lines, an average of 0.041% of the total MS1 signal intensity was from human-unique peptides in EVs isolated from PDX-bearing mice, compared with only 0.001% in plasma EVs from control nontumor-bearing mice. Breast tumors had the highest amount of tumor-derived protein, greater than 0.05% of all EV proteins detected (Fig. 1*C*). PDAC tumors produced the fewest tumor-derived EV proteins, on average 0.02% of all EV proteins. PDXs derived from metastatic sites released more EV protein than PDXs derived from primary sites (Fig. 1*D*), consistent with the known role of EVs in metastasis and formation of the metastatic niche (1, 2, 3).

The packaging of proteins into EVs by individual PDXs was consistent across biological replicates. For example, while the overall amount of tumor-derived proteins in EVs varied widely across the entire PDX set, they were similar within biological replicates of each PDX (Fig. 1*E*). A heatmap of human protein expression demonstrated that all biological replicates for 11 of the 14 PDX clusters next to each other (Fig. 1*F*). Statistically, the Spearman correlation (*r*^2^) of tumor-derived proteins within replicates of individual PDXs was 0.76, significantly higher than across the PDX cohort (0.42, *p* < 0.001). The consistent packaging of proteins into EVs by individual PDXs likely reflects the intrinsic biology of each tumor, but it is also possible that these patterns are associated with certain molecular subtypes.

We next determined if the tumor-derived EV proteome could be used to classify the underlying cancer present. The open source automated machine learning Python library Auto-Sklearn 2.0 (29) was used to generate models, and standard training and “holdout” testing methodology assessed accuracy (33). To avoid overfitting, we used only the LDA classifier, which predicted the underlying tumor type with greater than 92% accuracy. As shown in Figure 2, the single LDA classification algorithm is capable of a high degree of separation of tumor types and metastatic origin based on the tumor-derived EV proteome.

One caveat of this classification analysis is that while PDAC and breast cancers were a mix of primary tumors and those of metastatic origin, all CRC and melanoma PDXs were of metastatic origin (supplemental Table S1). It is also possible that the site of PDX implantation plays a role, since the breast PDXs were orthotopic, whereas other PDX models were implanted subcutaneously. However, application of this cross-species proteomics method to additional cancer types and larger xenograft cohorts will likely reveal further insight into the EV biology of cancer subtypes and metastasis.

### Expression Patterns of Tumor-Derived Proteins and Their Roles in EVs

**Fig. 3:**
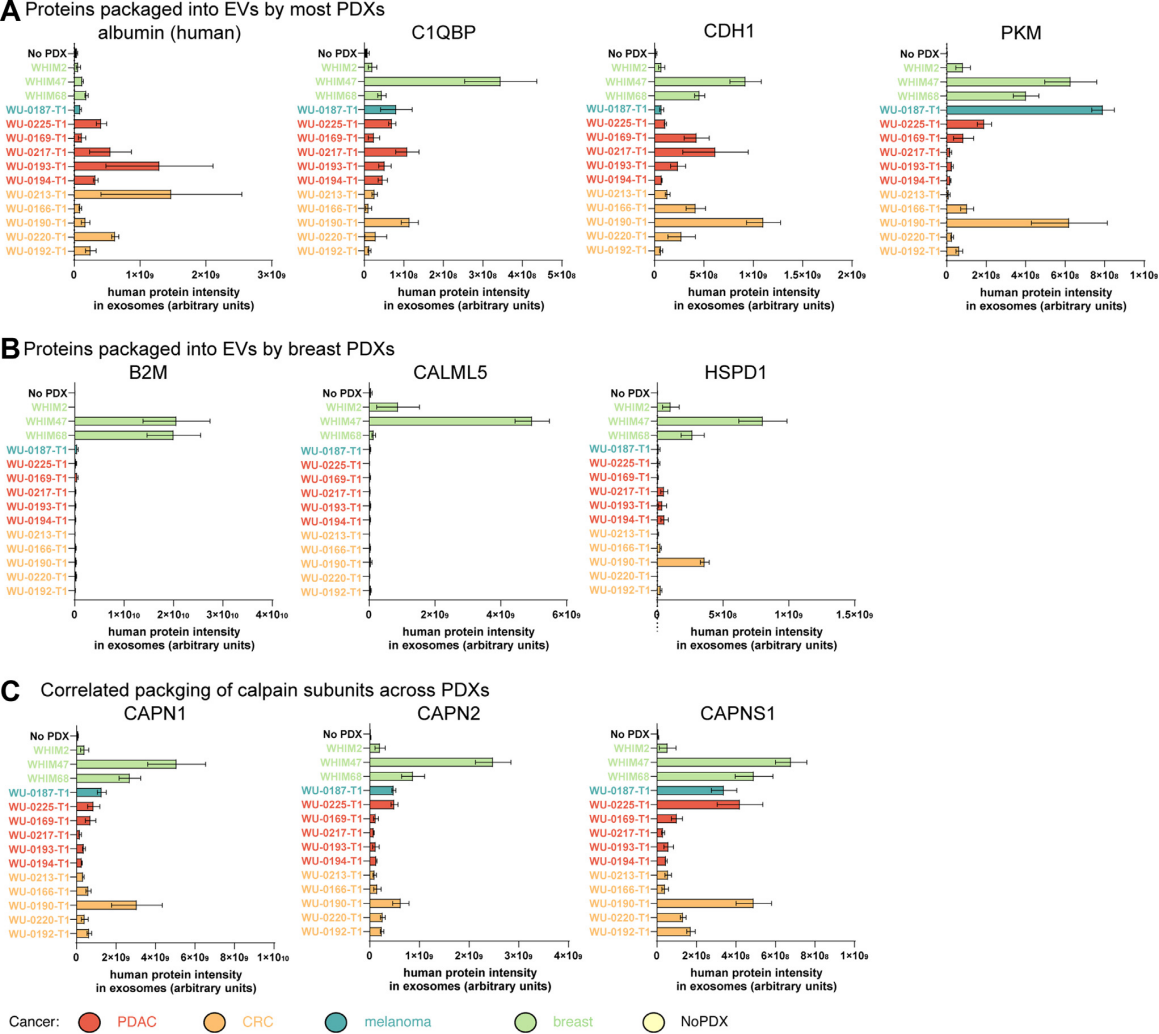
**Abundance of tumor-derived proteins in EVs reveals pan-cancer markers and cancer-unique indicators.***A*, relative human protein abundance of newly identified and known components of cancer EVs that pan-cancer markers present in most tumor-derived EVs. *B*, human proteins detected from EVs specific for breast cancer. *C*, relative human protein abundance of members of the calpain protein complex in EVs. N is detailed in supplemental Table S1. EV, extracellular vesicle.

Investigating the packaging of individual proteins revealed new insights into tumor-derived EVs *in vivo*. For example, several proteins were pan-cancer markers present in EVs from nearly all PDX models, including ALB, C1QBP, CDH1, and PKM (Fig. 3*A*). CDH1 has been shown to localize to the surface of tumor EVs and to heterodimerize with vascular endothelial-cadherin on the surface of endothelial cells to promote signaling (34). While previous studies utilized ovarian and prostate cell lines *in vivo*, these results demonstrate that patient-derived tumors of many cancer types generate CDH1-positive EVs.

Albumin's expression in EVs is not well characterized, primarily because albumin is typically filtered out as a nonspecifically bound contaminant. Since our method can distinguish the origin of the albumin, it is able to provide new insight into tumor-dependent packaging of albumin into EVs. We previously confirmed that albumin is expressed in several of these PDX models using cross-species proteomics (16) and thus is available for packaging by the tumor. It has also been shown that albumin is found in distinct EV subpopulations (35). Furthermore, lymphoma and leukemia cell lines package albumin into small and lipid-like entities that are likely EVs, which inhibit T-cell activation, proliferation, and function (36). Since albumin is an abundant component of nearly all PDX-derived EVs, its immunosuppressive role in EVs (36) may have unanticipated molecular and clinical importance.

The packaging of some proteins was limited to certain cancer subtypes, especially breast cancer (Fig. 3*B*). For example, B2M, CALML5, and HSPD1 are largely breast cancer–specific markers, but even these differ in their expression across the different three breast cancer PDXs. Many other proteins thought to be highly abundant in EVs also did not have a pan-cancer expression, including GAPDH, HSPD1, and ENO1 (supplemental Fig. S3).

Tumor-derived proteases in EVs facilitate remodeling of the extracellular matrix to promote the metastatic niche (1). Therefore, we noted the presence of MMP7 and three calpain components, CAPN1, CAPN2, and CAPNS1, as tumor-derived EV protein cargo (Figs. 3*C* and S3). While calpains are not well characterized in EVs, the highly correlated expression profiles of the three calpain component across the 14 PDXs suggest that these known interaction partners (37) are likely packaged into EVs together preconfigured as a complex (Fig. 3*C*). In contrast, MMP7 had very different expression pattern, which was almost nonoverlapping with calpains (supplemental Fig. S3). This indicates that cancers package proteases into EVs in a highly individualized manner, which may explain their varied effect on the ECM (1) and also provide new molecular targets to inhibit formation of the metastatic niche and metastasis.

## Discussion

We report a new cross-species proteomic approach to detect *bona fide* tumor-derived proteins in plasma EVs. This method uses commercial EV capture reagents and open source software, thus it can be broadly applied to assay cancer EVs in xenograft models. We demonstrate that this approach can identify *bona fide* tumor-derived proteins *in vivo*, including new protein complexes, and allows classification of the cancer type of the underlying PDX tumor with greater than 92% accuracy.

Plasma EVs are frequently used for biomarker studies because of their easy accessibility and the important mechanistic role they play in cancer. However, the EVs found in plasma can be derived from nearly any tissue, not just those associated with disease. This leads to challenges of interpreting plasma EV profiling results, since the tissue of origin is unknown. It also leads to sensitivity challenges, since the tissue or tumor may not produce many exosomes. Our approach provides clear assessment into the tumor origin, but it has several challenges to consider. First, the sensitivity is directly related to EV production by the tumor, a challenge shared by all plasma EV approaches. Second, there is significant species conservation between mouse and human, which limits the number of human-unique peptides. For example, 35% of identified peptides have sequences shared between human and mouse. Together, this resulted in only 2.2% of the identified peptides being assigned to human. On one hand, this limits the sensitivity of the method, but we are able to clearly demonstrate that most plasma EVs are not derived from the tumor, at least in PDX models. This result suggests that many cancer biomarkers in patients may be derived from the stroma as opposed to the tumor itself, though further investigation is needed.

PDXs are gaining popularity to study cancer and can be used to model cancer growth, metastasis, and drug response (38, 39). Further application of this EV proteomics approach could provide new insights into the basic biology and diagnostic utility of cancer-derived EVs by defining new cancer biomarkers, determining how patient tumors are responding to anticancer therapies, and delineating drug resistance mechanisms.

## Data Availability

The proteomic results have been deposited to ProteomeXchange consortium with the dataset ID of PXD028662. To view these data, follow the link at the ProteomeXchange page for the dataset to UCSD’s Massive and login as the site instructs using username: MSV000088128_reviewer and password: Held_005. Public access will become available once the article is accepted.

The experiment has been uploaded to EV-Track with entry ID EV220125. This may be accessed *via* the following EV-TRACK URL: http://evtrack.org/review.php. Please use the EV-TRACK ID (EV220125) and the last name of the first author (Barlin) to access our submission.

## Code Availability

A viewable Jupyter lab notebook used for data analysis is included as a supplemental file DataAnalysisCode.pdf.

## Supplemental data

This article contains supplemental data.

## Conflict of interest

Dr. Li has received license fee from Envigo and research funding from 10.13039/100004319Pfizer, 10.13039/100011957Takeda Oncology, and Zenopharm not associated with this article.

## Supplemental Data

Fig. S3

TableS1

TableS2

TableS3

MISEV2018_Checklist

DataAnalysisCode
