# Supplementary figures and images for: Proteins in Tumor-Derived Plasma Extracellular Vesicles Indicate Tumor Origin
Source: Mol Cell Proteomics. 2022 Dec 5;22(1):100476. doi: 10.1016/j.mcpro.2022.100476 (PMC9801135; doi:10.1016/j.mcpro.2022.100476)

# EV DLS

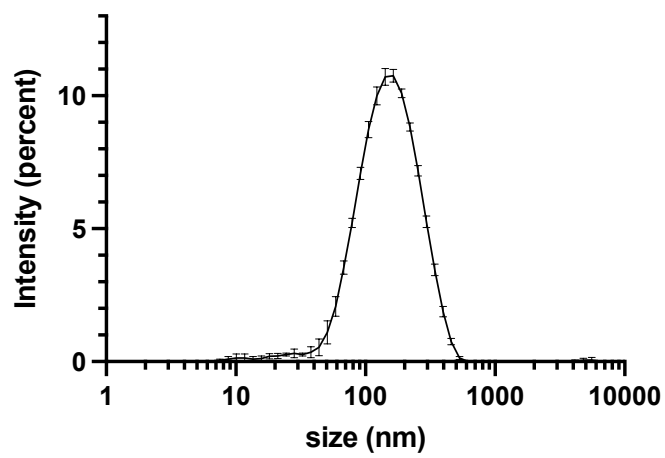

Supplement: Fig. S1 [file mmc1.pdf]

## Slide 1
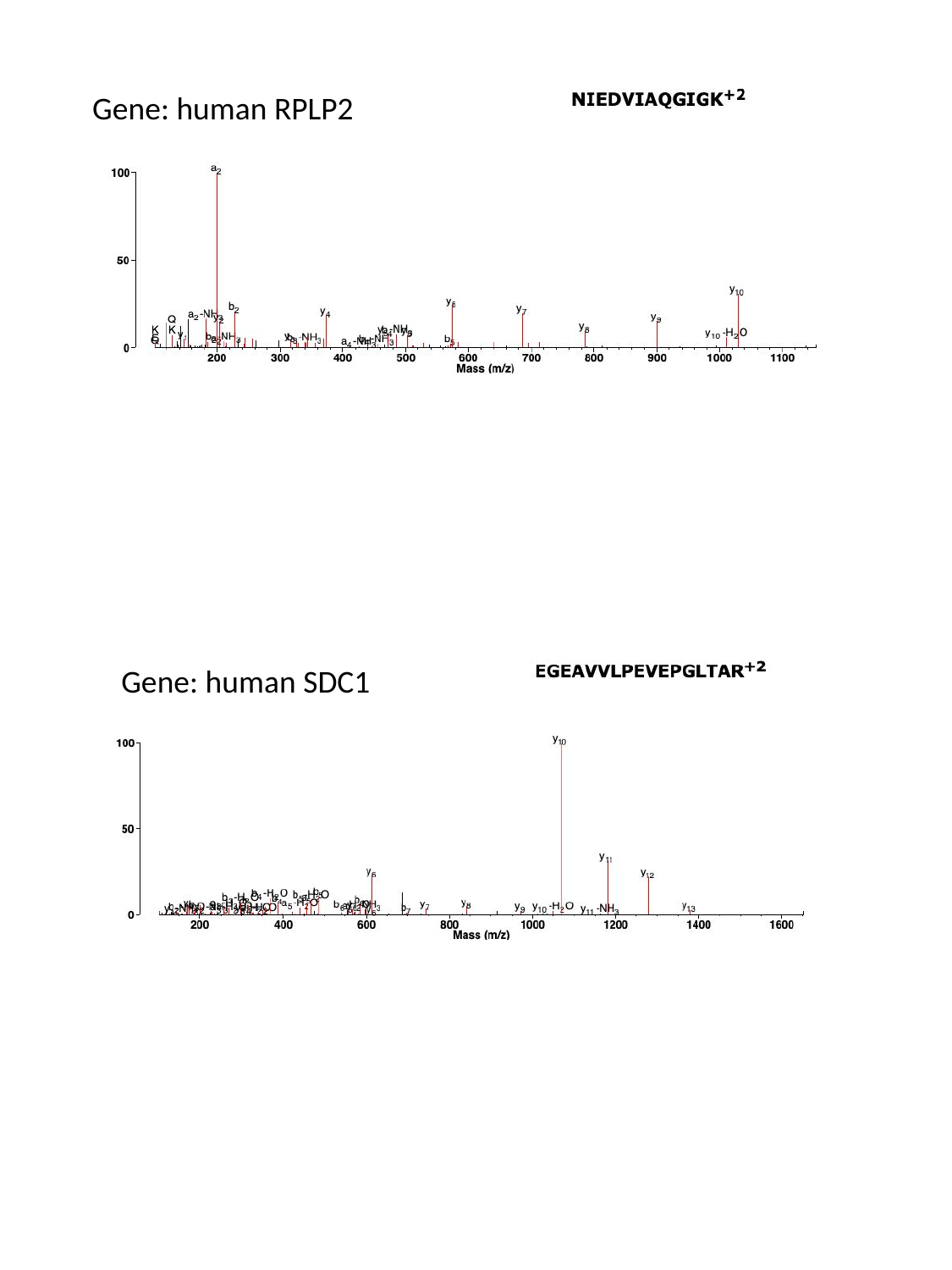

Gene: human RPLP2
Gene: human SDC1

Supplement: Fig. S2 [file mmc2.pptx]

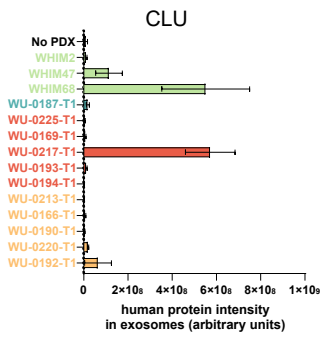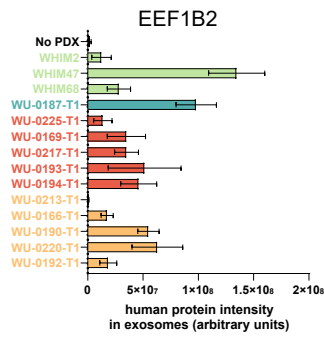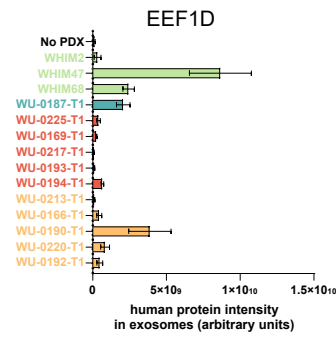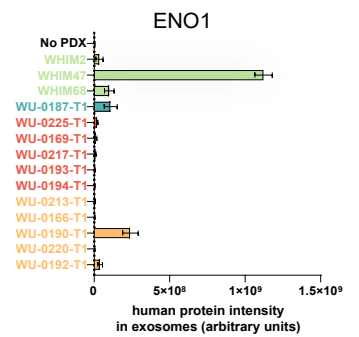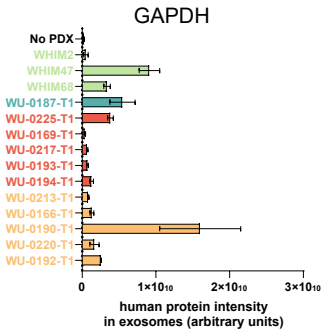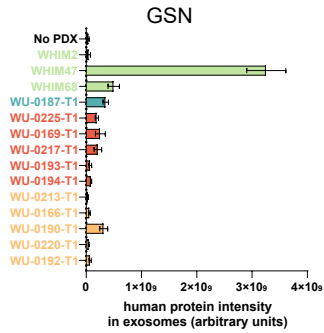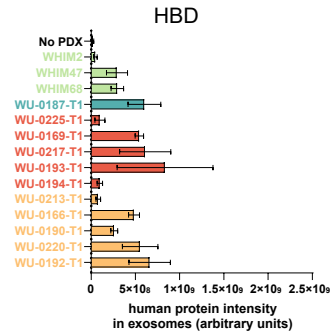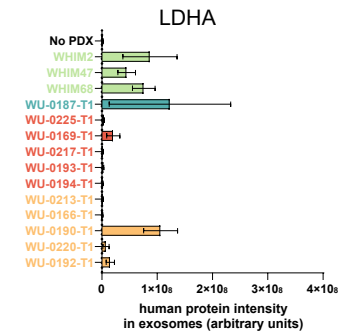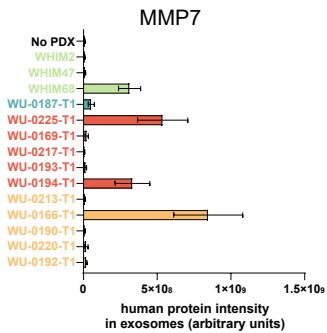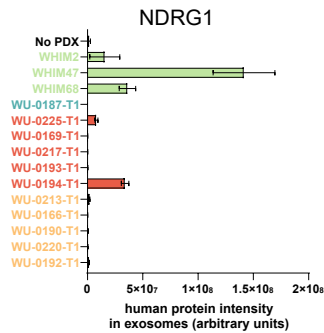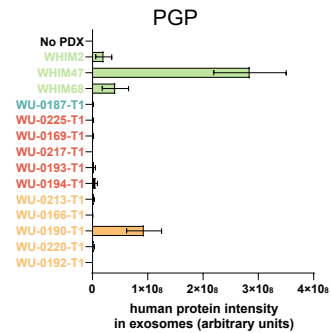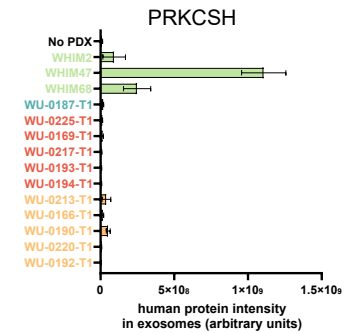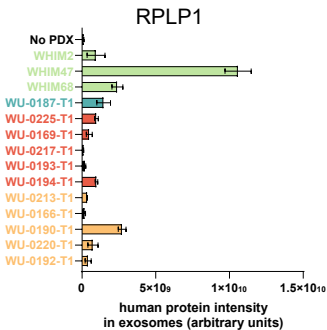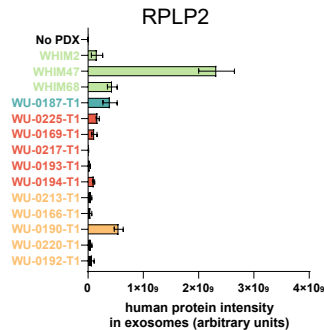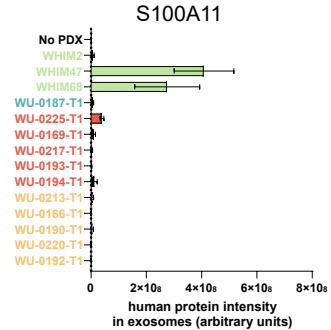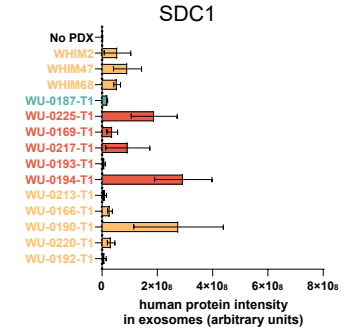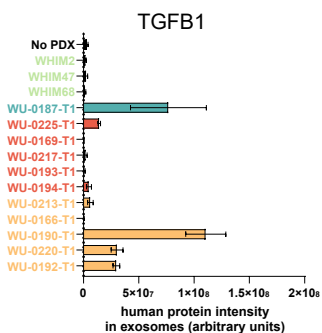

Cancer:  PDAC  CRC  melanoma  breast  NoPDX

Supplement: Fig. S3 [file mmc3.pdf]
