# Supplementary material for: Proteins in Tumor-Derived Plasma Extracellular Vesicles Indicate Tumor Origin
Source: Mol Cell Proteomics. 2022 Dec 5;22(1):100476. doi: 10.1016/j.mcpro.2022.100476 (PMC9801135; doi:10.1016/j.mcpro.2022.100476)
Supplement: MISEV2018_Checklist [file mmc7.docx]

**MISEV2018 Checklist**
*From C. Théry and K.W.Witwer, et al, ”Minimal Information for Studies of Extracellular Vesicles 2018 (MISEV2018): a position statement of the International Society for Extracellular Vesicles and update of the MISEV2014 guidelines”, J Extracell Vesicles 2018;7:1535750.*

+++ Mandatory

++ Mandatory if applicable

+ Encouraged

**1. Nomenclature**

*Mandatory*
✓ Generic term extracellular vesicle (EV): With demonstration of extracellular (no intact cells) and vesi-
cular nature per these characterization (Section 4) and function (Section 5) guidelines

*Encouraged (choose one)*

✓ Generic term extracellular vesicle (EV) + specification(size, density, other)

**2. Collection and pre-processing**

*Biofluids or Tissues (Sections 2-b and -c)*✓ Donor status if available (age, sex, food/water intake, collection time, disease, medication, other)
✓+++ Volume of biofluid or volume/mass of tissue sample collected per donor
✓++ Total volume/mass used for EV isolation (if pooled from several donors)
✓+++ All known collection conditions, including additives, at time of collection
✓ +++ Pre-treatment to separate major fluid-specific contaminants before EV isolation
✓+++ Temperature and time of biofluid/tissue handling
before and during pre-treatment
NA ++ For direct tissue EV extraction: treatment of tissue to release vesicles without disrupting cells

*Storage and recovery (Section 2-d)*
✓+++ Storage and recovery (e.g., thawing) of CCM, biofluid, or tissue before EV isolation (storage tempera-
ture, vessel, time; method of thawing or other sample preparation)
✓+++ Storage and recovery of EVs after isolation (temperature, vessel, time, additive(s)...)

**3. EV separation and concentration**

*Experimental details of the method*
✓++ Centrifugation: reference number of tube(s), rotor(s), adjusted k factor(s) of each centrifugation step (= time+speed+ rotor, volume/density of centrifugation conditions), temperature, brake settings

- Note, we include relative speed (x g) as an alternative to k factors

NA ++ Density gradient: nature of matrix, method of generating gradient, reference (and size) of tubes, bottom-
up (sample at bottom, high density) or top-bottom (sample on top, low density), centrifugation speed and
time (with brake specified), method and volume of fraction recovery

NA++ Chromatography: matrix (nature, pore size,...), loaded sample volume, fraction volume, number

✓++ Precipitation: reference of polymer, ratio vol/vol or weight/vol polymer/fluid, time/temperature of incubation, time/speed/temperature of centrifugation

NA ++ Filtration: reference of filter type (=nature of membrane, pore size...), time and speed of centrifugation,
volume before/after (in case of concentration)

NA ++ Antibody-based : reference of antibodies, mass Ab/amount of EVs, nature of Ab carrier (bead, surface) and amount of Ab/carrier surface

✓ ++ Other...: all necessary details to allow replication

NA ++ Additional step(s) to concentrate, if any

✓++ Additional step(s) to wash matrix and/or sample, if any

*Specify category of the chosen EV separation/concentration method (Table 1):*
✓+ High recovery, low specificity = mixed EVs and non-EV components OR
NA + Intermediate recovery, intermediate specificity =mixed EVs with limited non-EV components OR
NA + Low recovery, high specificity = subtype(s) of EVs with as little non-EV as possible OR
NA + High recovery, high specificity = subtype(s) of EVs with as little non-EV as possible

**4. EV characterization**

*Quantification (Table 2a, Section 4-a).*
✓+++ Volume of fluid, and/or cell number, and/or tissue mass used to isolate EVs
✓+++ Global quantification by at least 2 methods: **protein amount**, particle number, **lipid amount**, expressed
per volume of initial fluid or number of producing cells/mass of tissue
✓+++ Ratio of the 2 quantification figures

*Global characterization (Section 4-b, Table 3).*✓+++ Transmembrane or GPI-anchored protein localized in cells at plasma membrane or endosomes

- Many examples for the reader to choose from are in the proteomic data, Table S2.

✓+++ Cytosolic protein with membrane-binding or association capacity

- Many examples for the reader to choose from are in the proteomic data, Table S2.

✓+++ Assessment of presence/absence of expected contaminants

- Many examples for the reader to choose from are in the proteomic data, Table S2.

*At least one each of the three categories above.*

✓++ Presence of proteins associated with compartments other than plasma membrane or endosomes
✓++ Presence of soluble secreted proteins and their likely transmembrane ligands
+ Topology of the relevant functional components (Section 4-d)

*Single EV characterization (Section 4-c)*
NA +++ Images of single EVs by wide-field *and* close-up: e.g. electron microscopy, scanning probe microscopy, super-resolution fluorescence microscopy

- We note that the two corresponding authors of the MISEV 2018 criteria, Drs. Kenneth W. Witwer and Clotilde Thery, do not image single EVs by wide field and close up in a 2019 proteomics-focused paper that similar to ours (<https://doi.org/10.15252/embj.2020105492>). Thus, we do not consider this requirement as applicable to our similar proteomics study. Notably, we assay plasma, which is more challenging and limiting than the cell line exosome used in the study we reference.

✓+++ Non-image-based method analysing large numbers of single EVs: NTA, TRPS, FCS, high-resolution flow cytometry, multi-angle light-scattering, Raman spectroscopy, etc.

**5. Functional studies**

Not applicable

**6. Reporting**

✓+ Submission of methodologic details to EV-TRACK (evtrack.org) with EV-TRACK number provided (strongly encouraged). EV-TRACK Number is EV220125.
✓+++ Submission of data (proteomic, sequencing, other) to relevant public, curated databases or open-access
repositories
+ Data submission to EV-specific databases (e.g., EVpedia, Vesiclepedia, exRNA atlas)
✓++ Temper EV-specific claims when MISEV requirements cannot be entirely satisfied (Section 6-b)

- The MISEV criteria are satisfied.
