# Supplementary material for: Proteins in Tumor-Derived Plasma Extracellular Vesicles Indicate Tumor Origin
Source: Mol Cell Proteomics. 2022 Dec 5;22(1):100476. doi: 10.1016/j.mcpro.2022.100476 (PMC9801135; doi:10.1016/j.mcpro.2022.100476)
Supplement: DataAnalysisCode [file mmc8.pdf]

Data analysis code

```
[ ]: import numpy as np
import pandas as pd
import matplotlib
import matplotlib.pyplot as plt
import chart_studio.plotly as py
import plotly.express as px
import plotly.graph_objects as go
from matplotlib import colors
import os
from numpy import ma
from plotly.subplots import make_subplots
import tkinter as tk
import seaborn as sns
pd.set_option('display.max_columns',200)
%matplotlib inline
import plotnine as p9
from plotnine import *
from plotnine.data import *
from mizani.formatters import scientific_format
import altair as alt
from sklearn.preprocessing import StandardScaler
from scipy import stats
from scipy.stats import zscore
from sklearn.decomposition import PCA
from platform import python_version
print(python_version())
from pprint import pprint
```

1 Step 1: Import & Inspect dataframes (dfRaw)

- join metadata and ProteoClade annotated MaxQuant results

```
[ ]: from tkinter.filedialog import askopenfilename

root = tk.Tk()
root.withdraw()
```

```
dfInputFilename = askopenfilename(title =[('Human+Mouse data, PARSED with␣
 ↪ProteoClade')])
metadata = askopenfilename(title =[('Metadata')])
```

[ ]:
```
#input dataframes
dfInput = pd.read_table(dfInputFilename, sep=',')
metadata = pd.read_excel(metadata)
```

[ ]:
```
# merge raw and metadata dataframes
dfMerged = pd.merge(dfInput, metadata, on = 'Raw file', how ='left')

# store
dfRaw = dfMerged
```

2 Step 2: Clean data (dfRaw -> dfClean)

2.1 Step 2a: remove unneeded columns and camelCase column names

[ ]:
```
df = dfRaw
```

[ ]:
```
# drop unneeded columns

colsToDrop = ['Deamidation (N) Probabilities', 'Oxidation (M) Probabilities',
       'Deamidation (N) Score diffs', 'Oxidation (M) Score diffs',
       'Acetyl (Protein N-term)', 'Deamidation (N)', 'Gln->pyro-Glu',
       'Oxidation (M)', 'Fragmentation', 'Mass analyzer', 'Type', 'Scan event␣
 ↪number',
       'Isotope index','Combinatorics',
       'PIF', 'Fraction of total spectrum', 'Base peak fraction',
       'Precursor full scan number', 'Number of matches', 'Intensity coverage',␣
 ↪'Peak coverage',
       'Neutral loss level', 'ETD identification type', 'All scores', 'All␣
 ↪sequences', 'All modified sequences', 'Reporter PIF',
       'Reporter fraction', 'id', 'Protein group IDs', 'Peptide ID',
       'Mod. peptide ID', 'Evidence ID', 'Deamidation (N) site IDs',
       'Oxidation (M) site IDs', 'Localization prob', 'Precursor apex␣
 ↪fraction', 'Precursor apex offset',
       'Precursor apex offset time', 'Intensities',
       'Mass deviations [Da]', 'Mass deviations [ppm]', 'Score diff', 'Masses',␣
 ↪'Matches']

dfMinCols = df.drop(columns=colsToDrop)
```

[ ]:
```
dfRenamed = dfMinCols.rename(
    columns={
        'Raw file': 'rawFile',
```

```
        'Scan number': 'scanNumber',
        'Scan index': 'scanIndex',
        'Sequence': 'sequence',
        'Length': 'peptideLength',
        'Missed cleavages': 'missedCleavages',
        'Modifications': 'modifications',
        'Modified sequence': 'modifiedSequence',
        'Proteins': 'proteinsMQ',
        'Gene Names': 'geneNamesMQ',
        'Protein Names': 'proteinNamesMQ',
        'Charge': 'charge',
        'm/z': 'mz',
        'Mass': 'mass',
        'Mass error [ppm]': 'massErrorPPM',
        'Mass error [Da]': 'massErrorDA',
        'Simple mass error [ppm]': 'simpleMassErrorPPM',
        'Retention time': 'retentionTime',
        'PEP': 'pep',
        'Score': 'score',
        'Delta score': 'deltaScore',
        'Precursor Intensity': 'precursorIntensity',
        'Reverse': 'reverse',
        'organisms': 'organisms',
        'genes': 'genes',
        'Cancer': 'cancer',
        'Sample': 'sample',
        'Replicate': 'replicate',
        'Gender': 'gender'
    }
)

dfClean = dfRenamed
```

[ ]:
```
# add 'sampleAndReplicate' name

df = dfClean

df['sampleAndReplicate'] = df['sample'] + df['replicate']

dfClean = df
```

2.2 Step 2b: PEP filter dataframes (-> dfSig)

[ ]:
```
# keep rows PEP less than 0.01

df = dfClean
```

```
dfClean = df[df['pep'] <0.01]
```

2.3 Step 2c: drop rows with no Precursor intensity

[ ]:
```
# drop rows with no Precursor intensity (in order to find human, later replace␣
↪these with 0)

df = dfClean

df = df.dropna(subset=['precursorIntensity'])

dfClean = df
```

2.4 Step 2d: Filter out Reverse hits

[ ]:
```
df = dfClean

df['reverse'].fillna("")
df = df[~(df['reverse'].str.contains('+', na=False, regex=False))]

dfClean = df
```

2.5 Step 2e: Filter for only mouse|human assignment (dfClean)

[ ]:
```
df = dfClean

df = df[(df['organisms'].str.contains("9606|10090"))]

dfClean = df
```

[ ]:
```
# Assign ['organisms'] human readable names

df = dfClean

df.loc[(df['organisms'].str.contains("9606") & ~df['organisms'].str.
↪contains("10090") == True), 'organisms'] = 'human'
df.loc[(df['organisms'].str.contains("10090") & ~df['organisms'].str.
↪contains("9606") == True), 'organisms'] = 'mouse'
df.loc[(df['organisms'].str.contains("9606") & df['organisms'].str.
↪contains("10090") == True), 'organisms'] = 'shared'

dfClean = df
```

2.6 Step 2F: Assign peptides as gene-shared or gene-unique, use only gene-unique for quant

[ ]:
```
df = dfClean.copy()

# make all gene names uppercase
df['genes'] = df['genes'].str.upper()
df['genes'] = df['genes'].str.split("|")

df['genes'] = df['genes'].apply(set).str.join(', ')

# indicate unique or not
df.loc[~(df['genes'].str.contains(",") == True), 'genesUnique'] = 'genesUnique'
df.loc[(df['genes'].str.contains(",") == True), 'genesUnique'] = 'genesShared'

dfClean =df
```

2.7 Step 2 End: Save dfClean

[ ]:
```
dfClean = dfClean.reset_index(drop = True)
dfClean.to_excel('dfClean.xlsx')
```

2.8 Step 3a: Remove peptides not detected 6 times

[ ]:
```
# remove Modified sequences not observed z times

df = dfClean

z = 6

df['modifiedSequenceCounts'] = df.
↪groupby(by='modifiedSequence')['modifiedSequence'].transform('count').
↪reset_index(drop=True)
df = df[df['modifiedSequenceCounts'] >= z]

dfFiltered = df # fill in
```

2.9 Step 3b: Remove peptides not detected in all bio reps of 2 or more samples

[ ]:
```
# Peptide must be detected in all 3 Replicates in at least 2 samples

y = 3 # number of biorep detections needed per sample
z = 2 # number of samples that detect the peptide in >= y Replicates

df = dfFiltered
```

```
# first determine how many unique Replicates per Sample the Modified sequence␣
↪is detected in
dfNumReplicatesDetected = df.
↪pivot_table(index=['modifiedSequence','sample','replicate'],␣
↪values='rawFile', aggfunc=pd.Series.nunique)
dfNumReplicatesDetected = dfNumReplicatesDetected.rename(columns = {'rawFile':
↪'modifiedSequenceDetected'})

# Second, count how many BioReps detected the Modified sequence
dfNumReplicatesDetectedB = dfNumReplicatesDetected.
↪pivot_table(index=['modifiedSequence','sample'],␣
↪values='modifiedSequenceDetected', aggfunc="count")
dfNumReplicatesDetectedB = dfNumReplicatesDetectedB.rename(columns =␣
↪{'modifiedSequenceDetected':'modifiedSequenceRepsDetected'})

# merge newly created dfs
df2 = pd.merge(df, dfNumReplicatesDetected, on =␣
↪['sample','modifiedSequence','replicate'], how='left')
df2 = pd.merge(df, dfNumReplicatesDetectedB, on =␣
↪['sample','modifiedSequence'], how='left')

# Mask out and remove the rows without >=y detections
mask = df2['modifiedSequenceRepsDetected'] >= y
df3 = df2[mask]

# count how many samples have >= y detections
temp = df3.pivot_table(index=['modifiedSequence'], values='sample', aggfunc=pd.
↪Series.nunique)
temp = temp.rename(columns = {'sample':'modifiedSequenceSamplesDetected'})
dfFilter = pd.merge(df3, temp, on = ['modifiedSequence'], how='left')

# Mask out and remove the rows without >z samples detected.
mask = dfFilter['modifiedSequenceSamplesDetected'] >= z
df = dfFilter[mask]

dfFiltered = df
```

2.10 Step 3c: Remove 'crapome' genes (https://www.nature.com/articles/nmeth.2557)

[ ]:
```
df = dfFiltered

crapome =['KRT1', 'KRT2', 'KRT3', 'KRT4', 'KRT5', 'KRT6', 'KRT7', 'KRT8',␣
↪'KRT9', 'KRT10', 'KRT11', 'KRT12', 'KRT13', 'KRT14', 'KRT15', 'KRT16',␣
↪'KRT17', 'KRT18', 'KRT19', 'KRT20', 'KRT21', 'KRT22', 'KRT23', 'KRT24']

df = df.loc[~df['genes'].str.contains('|'.join(crapome))]
```

```
dfFiltered = df
```

2.11 Step 3d: Remove human peptides that were removed after skyline analysis (below) due to poor peak quality.

[ ]:
```
df = dfFiltered

onlyHumanPeptidesToKeep = ['AAFTECCQAADK', 'AAGVNVEPFWPGLFAK', 'AALEDTLAETEAR',␣
 ↪'AFDQDGDGHITVDELR', 'AFVDFLSDEIK', 'AGALNSNDAFVLK', 'AGLEDLQVAFR',␣
 ↪'ALANVNIGSLICNVGAGGPAPAAGAAPAGGPAPSTAAAPAEEK', 'ASLEGNLAETENR', 'ASLENSLR',␣
 ↪'AVMDDFAAFVEK', 'CCAAADPHECYAK', 'CGVPDVAEYSLFPNSPK', 'CLQSGTLFR',␣
 ↪'DAEAWFTSR', 'DMETIGFAVYEVPPELVGQPAVHLK', 'DTANWLEINPDTGAISTR',␣
 ↪'DYPVVSIEDPFDQDDWGAWQK', 'EEMQSNVEVVHTYR', 'EGEAVVLPEVEPGLTAR',␣
 ↪'EILSVDCSTNNPSQAK', 'ELHINLIPNK', 'ESGCSFVLALMQK', 'ETMVTSTTEPSR',␣
 ↪'FADDQLIIDFDNFVR', 'FFGLPITGMLNSR', 'GFSLESCR', 'GHAYSVTGAEEVESNGSLQK',␣
 ↪'GNPTVEVDLFTSK', 'GQVPENEANVVITTLK', 'GQVVSLIR', 'GTFSQLSELHCDKLHVDPENFR',␣
 ↪'GVVQELQQAISK', 'HIADLAGNSEVILPVPAFNVINGGSHAGNK', 'HIYYITGETK',␣
 ↪'IISNASCTTNCLAPLAK', 'ILGATIENSR', 'ISSIQSIVPALEIANAHR', 'ISSPTETER',␣
 ↪'LDETDDPDDYGDR', 'LDIDSPPITAR', 'LISWYDNEFGYSNR', 'LLPQLTYLDGYDR',␣
 ↪'LNVTEQEK', 'LPPGEYVVVPSTFEPNK', 'LQAEIEGLK', 'LTLLAPLNSVFK',␣
 ↪'LVINGNPITIFQER', 'LYELIITR', 'MPCQLHQVIVAR', 'MPPYDEQTQAFIDAAQEAR',␣
 ↪'NECLEAGTLFQDPSFPAIPSALGFK', 'NIEDVIAQGIGK', 'NTGVISVVTTGLDR', 'QNQEYQR',␣
 ↪'RPCFSALEVDETYVPK', 'RPTELLSNPQFIVDGATR', 'SHCIAEVENDEMPADLPSLAADFVESK',␣
 ↪'SLLEGQEDHYNNLSASK', 'SPAGLQVLNDYLADK', 'TATESFASDPILYRPVAVALDTK',␣
 ↪'TGAQELLR', 'THYSNIEANESEEVR', 'TILTLTGVSTLGDVK', 'TPSAAYLWVGTGASEAEK',␣
 ↪'TVQSLEIDLDSMR', 'VEHSDLSFSK', 'VHTECCHGDLLECADDR', 'VNHVTLSQPK',␣
 ↪'VPTANVSVVDLTCR', 'VTLTSEEEAR', 'VTTVASHTSDSDVPSGVTEVVVK',␣
 ↪'WGDAGAEYVVESTGVFTTMEK', 'YLNQDYEALR', 'YSDESGNMDFDNFISCLVR']

toRemove = df.loc[(df['organisms'].str.contains('human') & ~df['sequence'].str.
 ↪contains('|'.join(onlyHumanPeptidesToKeep)))]

df = pd.concat([df, toRemove, toRemove]).drop_duplicates(keep=False)

dfFiltered = df
```

2.12 Step3 End - Save dfFiltered

[ ]:
```
dfFiltered = dfFiltered.reset_index(drop = True)
dfFiltered.to_excel("dfFiltered.xlsx")
```

2.13 Step 4: Make dfFinal by restoring df all values of rebust peptides.

```
[ ]: # First, make a list of the robustly detected peptides (human or mouse or␣
     ↪shared)
     df = dfFiltered

     dfNoDups = df.drop_duplicates(subset=['modifiedSequence'])
     sequencesNoDups = dfNoDups['modifiedSequence'] #this list of modified sequences␣
     ↪that meet all the filter criteria.

     sequencesNoDups.to_excel('dfFiltered_Human|Mouse_modifiedSequenceListNoDups.
     ↪xlsx', columns=['modifiedSequence'])
```

```
[ ]: list = sequencesNoDups

     # go back to dfClean and pull out these peptides prior to filtering
     dfOrig = dfClean

     dfNew = pd.DataFrame()

     for i in list:
         eachRow = dfOrig.loc[dfOrig['modifiedSequence'] == i]
         dfNew = dfNew.append(eachRow)

     dfFinal = dfNew
     dfFinalBackup = dfNew.copy(deep=True)
```

2.14 Step 4 End: Save dfFinal

```
[ ]: dfFinal = dfFinal.reset_index(drop = True)
     dfFinal.to_excel("dfFinal.xlsx")
     dfFinalBackup = dfFinal
```

2.15 Step 5: Find good human (only) peptides (dfFindHighQualityHuman): filter data based on # detections

- just find HQ human peptides once, which is why this section is marked out, as this code only need run once
- dfClean -> dfFindHighQualityHuman

```
[ ]: # drop rows with no Precursor intensity (in order to find human, later replace␣
     ↪these with 0)

     #df = dfClean

     # remove control tumors since I don't want to count them towards being high␣
     ↪quality
```

```
#dfFindHighQualityHuman = dfFindHighQualityHuman.
 ↪loc[dfFindHighQualityHuman['Sample'] != 'Female']
#dfFindHighQualityHuman = dfFindHighQualityHuman.
 ↪loc[dfFindHighQualityHuman['Sample'] != 'Male']
```

[ ]:
```
# remove Modified sequences not observed z times

#df = dfFindHighQualityHuman

#z = 6 # number of times the Modified sequence

#df['modifiedSequenceCounts'] = df.groupby(by='Modified sequence')['Modified␣
 ↪sequence'].transform('count')
#dfFindHighQualityHuman = df[df['modifiedSequenceCounts'] >= z]
```

[ ]:
```
#z = 2 #number of samples that detect the peptide in >= 3 Replicates

#df= df_FindHighQualityHuman

# first determine how many Replicates per Sample the Modified sequence is␣
 ↪detected in
#df_NumReplicatesDetected = df.pivot_table(index=['Modified␣
 ↪sequence','Sample'], values='Replicate', aggfunc='count', fill_value=0)␣
 ↪#fill value is for missing values
#df_NumReplicatesDetected = df_NumReplicatesDetected.rename(columns =␣
 ↪{'Replicate':'Num Replicates Detected'})

#merge newly created df
#df = pd.merge(df, df_NumReplicatesDetected, on = ['Sample','Modified␣
 ↪sequence'], how='left')

#Second, find Modified sequences detected in all Replicates of at least z␣
 ↪samples
#grouped = df.groupby(['Sample','Modified sequence'])
#df['Num Replicates Detected'] = grouped.filter(lambda x: x['Num Replicates␣
 ↪Detected'].count() >= z)
#df_clean_filtered=df.dropna(subset=['Num Replicates Detected'])
#df_clean_filtered=df_clean_filtered.drop(columns=['Num Replicates Detected'])

#now pivot to calc how many samples have enough bioreps
#df_NumSamplesDetected = df_clean_filtered.pivot_table(index=['Modified␣
 ↪sequence'], values='Sample', aggfunc='count', fill_value=0) >z
#df_NumSamplesDetected = df_NumSamplesDetected.rename(columns = {'Sample':
 ↪'Samples All Bioreps Detected'})

#merge newly created df
```

```
#df_clean_filtered = df_clean_filtered.merge(df_NumSamplesDetected, left_on =␣
↪['Modified sequence'], right_on = ['Modified sequence'], how='left')

# Mask out and remove the rows without 4 samples
#mask = df_clean_filtered['Samples All Bioreps Detected'] == True
#temp = df_clean_filtered[mask] # whole big table with only the robust peptides
#temp2 = temp.drop_duplicates(subset=['Modified sequence'])

# This is the final list
#Modified_sequences_robust = temp2['Modified sequence'] #this list of modified␣
↪sequences that meet all the filter criteria.
```

2.16 Step 5b: Quantify human peptides with Skyline. See paper doi: 10.1093/bioinformatics/btq054

3 Step 6 is omitted for the purposes of this document

4 Step 7: Normalization (dfFinal -> dfNorm)

- Use dfFinal. dfFiltered is too stringently filtered by sample for this analysis.

[ ]:
```
df = dfFinal

dfNorm = df
```

4.1 Step 7.1: Plot species ratio per sample (doesn't need normalized)

[ ]:
```
# Plot species ratio per sample

df = dfNorm

grouping = 'sampleAndReplicate'

dfMouse = df.loc[df['organisms'].str.contains('mouse')].reset_index(drop=True)␣
↪# use to look up a specific species
dfHuman = df.loc[df['organisms'].str.contains('human')].reset_index(drop=True)␣
↪# use to look up a specific species
dfShared = df.loc[df['organisms'].str.contains('shared')].reset_index(drop=True)
dfMouse['IntensitySumSpecies'] = dfMouse['precursorIntensity'].
↪groupby(dfMouse[grouping]).transform(sum)
dfHuman['IntensitySumSpecies'] = dfHuman['precursorIntensity'].
↪groupby(dfHuman[grouping]).transform(sum)
dfShared['IntensitySumSpecies'] = dfShared['precursorIntensity'].
↪groupby(dfShared[grouping]).transform(sum)

#plot
fig = plt.figure(figsize=(25,10))
```

```
plt.subplots_adjust(wspace = 0.25, hspace = 0.25)

coord1=131
coord2=132
coord3=133

plt.subplot(coord1)
dfMouse.groupby(grouping)['IntensitySumSpecies'].sum().plot(kind='barh',␣
↪title='Mouse-unique');

plt.subplot(coord2)
dfHuman.groupby(grouping)['IntensitySumSpecies'].sum().plot(kind='barh',␣
↪title='Human-unique');

plt.subplot(coord3)
dfShared.groupby(grouping)['IntensitySumSpecies'].sum().plot(kind='barh',␣
↪title='Species-shared');
```

4.2 Step 7.2: Calculate total precursorIntensity per rawFile

[ ]:
```
df = dfNorm
grouping = ['rawFile']
yAxis = 'precursorIntensity'

for i in grouping:
    word1 = i
    capitalI = word1[0].upper() + word1[1:]
    word2 = yAxis
    capitalYAxis = word2[0].upper() + word2[1:]
    df['Total' + capitalYAxis + capitalI] = df.groupby([i])[yAxis].
↪transform('sum')
    df = df.reset_index()

dfNorm = df
```

4.3 Step 7.3a: Normalize each precursorIntensity by total rawFile signal

[ ]:
```
df = dfNorm

df['precursorIntensityFracRawFileIntensity'] = df['precursorIntensity']/
↪df['TotalPrecursorIntensityRawFile']

dfNorm = df
```

4.4 Step 7.3b: Normalize each precursorIntensity using Zscore

```
[ ]: df = dfNorm

# groupby and calc
df['precursorIntensityRawFileMean'] = df.
 ↪groupby(['rawFile'])['precursorIntensity'].transform('mean')
df['precursorIntensityRawFileStdev'] = df.
 ↪groupby(['rawFile'])['precursorIntensity'].transform('std')
df['precursorIntensityZScore'] =␣
 ↪(df['precursorIntensity']-df['precursorIntensityRawFileMean'])/
 ↪df['precursorIntensityRawFileStdev']

dfNorm =df
```

4.5 Step 7.4: Pivot by gene and impute 0 (dfNorm -> dfNormPivoted)

```
[ ]: # The normalized peptide precursor intensities above. Need to roll up to gene␣
 ↪level.

df = dfNorm

colsToDrop = ['rawFile', 'scanNumber', 'scanIndex', 'sequence',␣
 ↪'peptideLength', 'missedCleavages', 'modifications', 'modifiedSequence',
      'proteinsMQ', 'geneNamesMQ', 'proteinNamesMQ', 'charge', 'mz', 'mass',␣
 ↪'massErrorPPM', 'massErrorDA', 'simpleMassErrorPPM', 'retentionTime', 'pep',␣
 ↪'score', 'deltaScore', 'precursorIntensity', 'reverse', 'replicate',␣
 ↪'genesUnique', 'modifiedSequenceCounts', 'TotalPrecursorIntensityRawFile',␣
 ↪'precursorIntensityRawFileMean',
    'precursorIntensityRawFileStdev', 'precursorIntensityZScore']

df = df.drop(columns=colsToDrop)

# list all non-gene/quant columns
indexCols = ['sampleAndReplicate', 'cancer', 'sample','organisms',␣
 ↪'metastaticTumor','metTissue','metPDX']
colToPivot = 'genes'
quantColumn = 'precursorIntensityFracRawFileIntensity' # the pivot index

#pivot on quant column #sums genes, imputes 0 if missing
dfNormPivoted = df.pivot_table(index=indexCols, columns=colToPivot,␣
 ↪values=quantColumn, aggfunc=sum, fill_value=0)
dfNormPivoted = dfNormPivoted.reset_index('sampleAndReplicate').reset_index()

#add a TotalNormIntensityBySpecies column.
dfNormPivoted['TotalNormIntensityBySpecies'] = dfNormPivoted.sum(axis=1) # sum␣
 ↪row-wise across each column
```

```
dfNormPivoted.index.names = ['index']

dfNormPivoted.to_excel("dfFinalPivoted.xlsx")
```

[ ]:
```
# sum intensities for each [RAW file/sample].

df = dfNormPivoted
grouping = ['sampleAndReplicate', 'cancer', 'organisms']
yAxis = 'TotalNormIntensityBySpecies'

df.append(df.sum(numeric_only=True), ignore_index=True)
for i in grouping:

    Title = ("Total " + yAxis + " of Mouse|Human peptides per " + i)

    dfTemp = df.groupby([i])[yAxis].sum()
    dfTemp = dfTemp.reset_index()

    # to save
    dfTemp.to_excel(Title + '.xlsx', columns=[i, yAxis])
```

4.6 Step 9: PCA from Skyline (human only) results (dfSkyline -> dfPCA)

analysis steps

- 9.1 remove gene-shared peptides
- 9.2 curate down to the minimal columns
- 9.3 Use pivot table to ranspose the dataframe so that each row is a single sampleAndReplicate and each column is a gene and also impute 0 to make tidy data. Pivot by gene
- 9.4 Scale values to zero mean and unit varaince

[ ]:
```
from sklearn.decomposition import PCA
from sklearn.cluster import KMeans
from sklearn.manifold import TSNE
from sklearn.preprocessing import StandardScaler

import plotly.graph_objs as go
import plotly.offline as offline
offline.init_notebook_mode()
```

4.7 Step 9.0: import dfSkylineUnmelted

[ ]:
```
dfSkyline = pd.read_excel('/Users/jasonheld/Manuscripts/2021_PDX-exosomes/
 ↪Held005-Human-MS1.xlsx', sheet_name='dfSkylineUnmelted')
```

4.8 Step 9.1: remove gene-shared peptides

- performed as above

4.9 Step 9.2 re-scale human peptides dfSkyline

[ ]:
```
df = dfSkyline

# slice off the categorical names
colsToDrop = ['rawFile', 'cancer', 'sample', 'replicate', 'gender',␣
 ↪'mouseBackground','organisms','sampleAndReplicate','metTissue','metastaticTumor',␣
 ↪'metPDX']
dfSkylineForPCA = df.drop(colsToDrop, axis=1)

# column labels
dfTemp=dfSkyline.reset_index()
dfSkylineLabels=dfTemp.loc[:, ['cancer', 'organisms',␣
 ↪'sample','metastaticTumor','metTissue','metPDX']].reset_index(drop=True)
dfSkylineLabels

scaler = StandardScaler()

dfSkylineForPCAScaled = scaler.fit_transform(dfSkylineForPCA)

# save
dfSkylineForPCAScaledTemp = pd.DataFrame(dfSkylineForPCAScaled)
dfSkylineForPCAScaledTemp.to_excel('dfSkylineForPCAScaled.xlsx')
dfSkylineLabels.to_excel('dfSkylineLabels.xlsx')
```

4.10 Step 9.3: Perform PCA and project

[ ]:
```
# use scaled data

from sklearn.decomposition import PCA

scaledData = dfSkylineForPCAScaled

components = 6

skylinePCAOut= PCA(n_components=21).fit(scaledData)
skylineVarExp = skylinePCAOut.explained_variance_ratio_
skylineCumVarExp = np.cumsum(skylinePCAOut.explained_variance_ratio_)

skylinePCAOutFitTransformed = PCA(n_components=components).fit(scaledData).
 ↪transform(scaledData)
```

[ ]:
```
loadings = skylinePCAOut.components_

pcList = ['PC1', 'PC2', 'PC3', 'PC4', 'PC5', 'PC6']

skylineLoadingsDf = pd.DataFrame.from_dict(dict(zip(pcList, loadings)))
```

```
skylineLoadingsDf['variable'] = dfSkylineForPCA.columns.values
skylineLoadingsDf = skylineLoadingsDf.set_index('variable')
skylineLoadingsDf
```

4.11 Step 9: Effect of variables on each components

[ ]:
```
ax = sns.heatmap(skylinePCAOut.components_,
                 cmap='YlGnBu',
                 yticklabels=[ "PCA"+str(x) for x in range(1,skylinePCAOut.
 ↪n_components_+1)],
                 xticklabels=dfSkylineForPCA.columns.values,
                 cbar_kws={"orientation": "horizontal"})

ax.set_aspect("equal")

sns.set(rc={'figure.figsize':(6,6)})
```

4.12 Step 9: Component loadings plot

[ ]:
```
df = skylineLoadingsDf.reset_index()

Title = 'TempTitle'

# melt to unpivot the data for plotnine
df = df.melt(id_vars = 'variable', var_name ='principalComponent',␣
 ↪value_name='componentLoading')

#pull out the gene (e.g. variables) with the most extreme PCs to label
highestPC = df.sort_values(by='componentLoading', ascending=False).
 ↪groupby('principalComponent').nth(0).reset_index()
secondHighestPC = df.sort_values(by='componentLoading', ascending=False).
 ↪groupby('principalComponent').nth(1).reset_index()
thirdHighestPC = df.sort_values(by='componentLoading', ascending=False).
 ↪groupby('principalComponent').nth(2).reset_index()

lowestPC = df.sort_values(by='componentLoading').groupby('principalComponent').
 ↪nth(0).reset_index()
secondLowestPC = df.sort_values(by='componentLoading').
 ↪groupby('principalComponent').nth(1).reset_index()
thirdLowestPC = df.sort_values(by='componentLoading').
 ↪groupby('principalComponent').nth(2).reset_index()

(
ggplot(df,
       aes(x='principalComponent',
           y='componentLoading'))
```

```
    +geom_point(position='jitter', alpha=0.25)
    +geom_text(aes(x='principalComponent', y='componentLoading',␣
 ↪label='variable'), nudge_y=0.03, data=highestPC, size=10)
    +geom_text(aes(x='principalComponent', y='componentLoading',␣
 ↪label='variable'), nudge_y=0.02, data=secondHighestPC, size=10)
    +geom_text(aes(x='principalComponent', y='componentLoading',␣
 ↪label='variable'), nudge_y=0.01, data=thirdHighestPC, size=10)
    +geom_text(aes(x='principalComponent', y='componentLoading',␣
 ↪label='variable'), nudge_y=-0.03, data=lowestPC, size=10)
    +geom_text(aes(x='principalComponent', y='componentLoading',␣
 ↪label='variable'), nudge_y=-0.02, data=secondLowestPC, size=10)
    +geom_text(aes(x='principalComponent', y='componentLoading',␣
 ↪label='variable'), nudge_y=-0.01, data=thirdLowestPC, size=10)
    +ggtitle(Title)
    +theme(
        panel_background=element_rect(fill='white'),
        axis_line_x=element_line(color='black'),
        panel_grid=element_blank(),
        panel_border=element_blank(),
        figure_size=(5, 5),
    )
)
```

[ ]:

```
df = skylineLoadingsDf.reset_index()
Title = 'TempTile'

# melt to unpivot the data for plotnine
df = df.melt(id_vars = 'variable', var_name ='principalComponent',␣
 ↪value_name='componentLoading')

#pull out the gene (e.g. variables) with the most extreme PCs to label
highestPC = df.sort_values(by='componentLoading', ascending=False).
 ↪groupby('principalComponent').nth(0)
secondHighestPC = df.sort_values(by='componentLoading', ascending=False).
 ↪groupby('principalComponent').nth(1)
thirdHighestPC = df.sort_values(by='componentLoading', ascending=False).
 ↪groupby('principalComponent').nth(2)

lowestPC = df.sort_values(by='componentLoading').groupby('principalComponent').
 ↪nth(0)
secondLowestPC = df.sort_values(by='componentLoading').
 ↪groupby('principalComponent').nth(1)
thirdLowestPC = df.sort_values(by='componentLoading').
 ↪groupby('principalComponent').nth(2)
thirdLowestPC
```

[ ]:
```
# print the explained variance

print('Variance explained by First PC =',
    np.cumsum(skylinePCAOut.explained_variance_ratio_ * 100)[0])

print('Variance explained by First 2 PCs =',
    np.cumsum(skylinePCAOut.explained_variance_ratio_ * 100)[1])

print('Variance explained by First 3 PCs =',
    np.cumsum(skylinePCAOut.explained_variance_ratio_ * 100)[2])

print('Variance explained by First 10 PCs =',
    np.cumsum(skylinePCAOut.explained_variance_ratio_ * 100)[9])
```

[ ]:
```
# get correlation matrix plot for loadings
ax = sns.heatmap(skylineLoadingsDf, annot=True, cmap='Spectral')
plt.show()
```

4.13 Step 9: basic 2 PC plot

[ ]:
```
plt.figure(figsize=(6,6))

hue = 'cancer'
markerStyle = 'metPDX'

color = "Spectral"

sns.scatterplot(x=skylinePCAOutFitTransformed[:, 0],
                y=skylinePCAOutFitTransformed[:, 1],
                hue=dfSkylineLabels[hue],
                palette=color,
                edgecolor='black',
                s=60,
                style=dfSkylineLabels[markerStyle],
               )

plt.title("First 2 PCs")
plt.xlabel('PC1')
plt.ylabel('PC2')

plt.xscale('symlog')
plt.yscale('symlog')

plt.legend(loc='center left', bbox_to_anchor=(1, 0.5))

plt.savefig('PCA_2PCs_human_SymLog_' + hue + '_' + markerStyle +'.jpg', dpi=500)
plt.savefig('PCA_2PCs_human_SymLog_' + hue + '_' + markerStyle +'.svg', dpi=500)
```

```
plt.savefig('PCA_2PCs_human_SymLog_' + hue + '_' + markerStyle +'.pdf', dpi=500)
```

4.14 Step 10: Human heatmap

4.15 Step 10.0: import dfSkyline heatmap

[ ]:
```
dfSkylineHeatMap = pd.read_excel('/Users/jasonheld/Manuscripts/
↪2021_PDX-exosomes/Held005-Human-MS1.xlsx',␣
↪sheet_name='heatmap_StandardScaled', header = [0,3])

#reset index to make gene the index
dfSkylineHeatMap = dfSkylineHeatMap.set_index(dfSkylineHeatMap.iloc[:,0],␣
↪drop=True)
dfSkylineHeatMap = dfSkylineHeatMap.iloc[:,1:]
dfSkylineHeatMap.index.name = 'genes'
```

4.16 Step 10.1: make heatmap

[ ]:
```
sns.clustermap(
    dfSkylineHeatMap,
    figsize=(12,7),
    metric = 'jensenshannon',
    linewidth=0.004,
    linecolor = 'grey',
    method='average',
    standard_scale=0,
    cmap='flare_r',
    dendrogram_ratio=(.02, .08),
    cbar_pos=(-.06, .4, .03, .2),
)

plt.savefig('dfSkylineHeatMap.svg')
```

5 11. Classify results using auto-sklearn

- https://automl.github.io/auto-sklearn/master/

[ ]:
```
import autosklearn.classification
import sklearn.model_selection
import sklearn.datasets
import sklearn.metrics
from autosklearn.experimental.askl2 import AutoSklearn2Classifier
```

[ ]:
```
# import data
dfSkyline = pd.read_excel('/Users/jasonheld/Manuscripts/2021_PDX-exosomes/
↪Held005-Human-MS1.xlsx', sheet_name='dfSkylineUnmelted')
```

```
dfSkyline.head()
```

[ ]:
```
df = dfSkyline

# For human only (e.g. don't comment out when using human)
# remove control tumors since I don't want to count them towards being high␣
↪quality/ != means not equal
#df = df.loc[df['sample'] != 'CTRLFemale'] # this filters out
#df = df.loc[df['sample'] != 'CTRLMale'] # this filters out

#df = df[(df['organisms'].str.contains(grouping))]

dfSkyline = df
```

5.1 Step 11.2 cancer classification

[ ]:
```
# import data
df = dfSkyline

colsToDrop = [
    'rawFile',
#     'cancer',
    'sample',
    'replicate',
    'gender',
    'mouseBackground',
    'organisms',
    'sampleAndReplicate',
    'metTissue',
    'metastaticTumor',
    'metPDX'
    ]
dfSkylineForLDA = df.drop(colsToDrop, axis=1)

# get column labels
dfTemp=dfSkyline.reset_index()
dfSkylineLDALabels=dfTemp.loc[:, ['cancer', 'organisms',␣
↪'sample','metastaticTumor','metTissue','metPDX']].reset_index(drop=True)
dfSkylineLDALabels

y = dfSkylineForLDA.iloc[:,0]
proteinHeader = dfSkylineForLDA.columns[1:]
X_all = dfSkylineForLDA.iloc[:,1:]
```

[ ]:
```
from sklearn.model_selection import train_test_split

#43,44 is 0.9 45 is 0.7
```

```
X_train, X_test, y_train, y_test = train_test_split(X_all, y, test_size=0.25,␣
↪random_state=1)

print(X_test[:5])
print(y_test[:5])
```

5.1.1 Feature Scaling

[ ]:
```
from sklearn.preprocessing import StandardScaler

# do all now
sc = StandardScaler()
X_train = sc.fit_transform(X_train)
X_test = sc.transform(X_test)
X_all = sc.fit_transform(X_all)

print(X_all[:5])
```

[ ]:
```
# define the search
automl = autosklearn.classification.AutoSklearnClassifier(
    include={
        'classifier': [
            'lda'
        ],
    },
    ensemble_size=1,
    n_jobs = 4,
        time_left_for_this_task=300,
        per_run_time_limit=30,
    metric=autosklearn.metrics.accuracy,
)

# perform the search
automl.fit(X_train, y_train)
y_hat = automl.predict(X_test)
print("Accuracy score (holdout)", sklearn.metrics.accuracy_score(y_test, y_hat))
```

6 Step 12: LDA (in R)

- note, this has to run using an R kernel

[ ]:
```
library(proteoQDA)
library(proteoQ)
library(NMF)
library(downloader)
library(RColorBrewer)
library(pheatmap)
```

```
library(ggplot2)
library(tidyverse)
library(ggthemes)
library(MASS)
library(ggrepel)
library(caret)
library(doMC)
`%notin%` <- Negate(`%in%`)
library(caretEnsemble)
library(doParallel)
library(dplyr)
library(PerformanceAnalytics)
library(corrplot)
library(car)
library(psych)
library(AppliedPredictiveModeling)
R.Version()
```

[ ]:
```
# import the python support file made above focused on LDA
setwd("/Users/jasonheld/Manuscripts/2021_PDX-exosomes/Python")
dfX_ally = read.csv(file = "dfX_ally_cancer.csv", stringsAsFactors = T)
dfX_all <- dfX_ally[, -which(names(dfX_ally) == "cancer")]
```

[ ]:
```
# Split samples based on the outcome
set.seed(111114)
trainIndex <- createDataPartition(dfX_ally$cancer, p = .4,
                                  list = FALSE,
                                  times = 1)

# split off and center/scale training and testing.
preProcValues <-preProcess(dfX_ally, method = c("center", "scale"))
dfX_ally <- predict(preProcValues, dfX_ally)

dfX_trainy <- dfX_ally[trainIndex,]
preProcValues <-preProcess(dfX_trainy, method = c("center", "scale"))
dfX_trainy <- predict(preProcValues, dfX_trainy)

dfX_testy  <- dfX_ally[-trainIndex,]
preProcValues <-preProcess(dfX_testy, method = c("center", "scale"))
dfX_testy <- predict(preProcValues, dfX_testy)
```

[ ]:
```
# Train
dfX_ally.lda <- lda(
    cancer ~.,
    data = dfX_ally,
    method = 'moment',
    )
```

[ ]:
```r
# Predict
dfX_ally.predicted.lda <- predict(dfX_ally.lda,
    newdata = dfX_ally,
    method = 'plug-in',
    )
```

[ ]:
```r
# Plot
newdata <- data.frame(type = dfX_ally[,1], lda = dfX_ally.predicted.lda$x)
ggplot(newdata) + geom_point(aes(lda.LD1, lda.LD2, colour = dfX_ally$cancer),␣
 ↪size = 2.5)+
  guides(fill = "none") +
  labs(title = "", x = "LD1", y = "LD2") +
  theme_minimal() +
  theme(aspect.ratio = 1)

ggsave(file.path("proteinLDA_ggplot_LD1-LD2_manual_cancer.pdf"))
```

[ ]:
```r
# plot LD2-LD3 if available based on # of comparisons

ggplot(newdata) + geom_point(aes(lda.LD2, lda.LD3, colour = dfX_ally$cancer),␣
 ↪size = 2.5)+
  guides(fill = "none") +
  labs(title = "", x = "LD2", y = "LD3") +
  theme_minimal() +
  theme(aspect.ratio = 1)

ggsave(file.path("proteinLDA_ggplot_LD2-LD3_manual_cancer.pdf"))
```

[ ]:
```r
# plot LD1-LD3 if available based on # of comparisons

ggplot(newdata) + geom_point(aes(lda.LD1, lda.LD3, colour = dfX_ally$cancer),␣
 ↪size = 2.5)+
  guides(fill = "none") +
  labs(title = "", x = "LD1", y = "LD3") +
  theme_minimal() +
  theme(aspect.ratio = 1)

ggsave(file.path("proteinLDA_ggplot_LD1-LD3_manual_cancer.pdf"))
```
